# Engineering Gold Shelled Nanomagnets for Pre-Setting the Operating Temperature for Magnetic Hyperthermia

**DOI:** 10.3390/nano12162760

**Published:** 2022-08-12

**Authors:** Elis Regina Lima Siqueira, Willie Oliveira Pinheiro, Victor Raul Romero Aquino, Breno Cunha Pinto Coelho, Andris Figueiroa Bakuzis, Ricardo Bentes Azevedo, Marcelo Henrique Sousa, Paulo Cesar Morais

**Affiliations:** 1 Department of Genetics & Morphology, Institute of Biological Sciences, University of Brasília, Brasília DF 70910-900, Brazil; 2 Green Nanotechnology Group, Faculty of Ceilândia, University of Brasília, Brasília DF 72220-900, Brazil; 3 Post-Graduation Program in Sciences and Health Technologies, Faculty of Ceilândia, University of Brasília, Brasília DF 72220-275, Brazil; 4 Institute of Physics, Federal University of Goiás, Goiânia GO 74690-631, Brazil; 5 Institute of Physics, University of Brasília, Brasília DF 70910-900, Brazil; 6 Federal Institute of Brasília, Brasília DF 24429-005, Brazil; 7 CNanoMed, Federal University of Goiás, Goiânia GO 74690-631, Brazil; 8 Catholic University of Brasília, Brasília DF 70790-160, Brazil

**Keywords:** magnetic hyperthermia, magnetic fluid, maghemite, core–shell, asymptotic temperature, Box–Lucas model

## Abstract

This study investigated the fabrication of spherical gold shelled maghemite nanoparticles for use in magnetic hyperthermia (MHT) assays. A maghemite core (14 ± 3 nm) was used to fabricate two samples with different gold thicknesses, which presented gold (g)/maghemite (m) content ratios of 0.0376 and 0.0752. The samples were tested in MHT assays (temperature versus time) with varying frequencies (100–650 kHz) and field amplitudes (9–25 mT). The asymptotic temperatures ($T_{\infty }$) of the aqueous suspensions (40 mg Fe/mL) were found to be in the range of 59–77 °C (naked maghemite), 44–58 °C (g/m=0.0376) and 33–51 °C (g/m=0.0752). The MHT data revealed that $T_{\infty }$ could be successful controlled using the gold thickness and cover the range for cell apoptosis, thereby providing a new strategy for the safe use of MHT in practice. The highest SAR (specific absorption rate) value was achieved (75 kW/kg) using the thinner gold shell layer (334 kHz, 17 mT) and was roughly twenty times bigger than the best SAR value that has been reported for similar structures. Moreover, the time that was required to achieve $T_{\infty }$ could be modeled by changing the thermal conductivity of the shell layer and/or the shape/size of the structure. The MHT assays were pioneeringly modeled using a derived equation that was analytically identical to the Box–Lucas method (which was reported as phenomenological).

## 1. Introduction

The principles that underlie magnetic hyperthermia (MHT) using magnetic nanoparticles (MNPs) have been extensively discussed over recent years [1,2,3]. In short, heat is dissipated mainly from the relaxation losses of the MNPs, which creates localized heat in the surrounding environment of the dispersed nanomagnets [4].

In cancer treatment, controlled localized heating with the temperature of the surrounding biological environment stabilized in the range of 41–48 °C represents a key strategy, which is challenging to achieve in practice but is nevertheless required to promote tumor cell death by apoptosis instead of necrosis. When the latter is triggered at higher temperatures, it has negative impacts on both the health of the patient and that of the local normal tissue [5]. Therefore, the use of this strategy within the medical field requires the surrounding environment to be at a safe temperature in order to promote apoptosis at the tumor site and avoid necrosis or any other adverse effects in the surrounding normal tissue, such as inflammation processes that are caused by untargeted overheating [6].

However, the lack of robust and accurate (preferably noninvasive) temperature probing methods for targeted biological sites has been a limiting factor in the application of MHT technology in practice [7]. Moreover, numerous MHT protocols have been employed in clinically unsafe conditions, which aimed to achieve therapeutic temperatures with minimal doses of the administrated MNPs [8].

Therefore, in MHT protocols, the strategy of pre-setting the asymptotic operating temperature ($T_{\infty }$) for the surrounding environment via the modulation of the morphological and magnetic properties of the MNPs, as well as the alternating magnetic field characteristics, is a very promising direction to explore. This approach could lead to important breakthroughs in the development of MHT technology from its current restricted status within clinical study to widespread clinical application. Moreover, regardless of the MHT protocol conditions, safe operation clearly requires the use of optimized biocompatible nanomagnets to deliver controlled heating outputs under low alternating magnetic field amplitudes [4,9]. Regarding heating control, different approaches have been reported in the literature, including the modulation of MNP concentrations within liquid media with varying viscosities [10], dipolar interaction via MNP clustering [11], the nonmagnetic metal doping of typical MNPs [12] and the use of core–shell magnetic nanostructures [13].

Surface dressed iron oxide NPs, such as maghemite (γ-Fe_2_O_3_), have been extensively employed for MHT purposes owing to their high colloidal stability when dispersed in aqueous media, superior biocompatibility and relatively well-known magnetic behavior for heat generation [14,15]. Additionally, metal shelled MNPs that use nonmagnetic metal shells (such as gold) to dress magnetic cores can improve both the magnetostatic and magnetodynamic properties of shelled nanomagnets [16]. Moreover, the metal shell layers can substantially improve the heat transfer between the magnetic iron oxide cores and their surrounding environment. Additionally, there is evidence that gold shells improve the biocompatibility of magnetic iron oxide nanoparticles [17,18,19]. Likewise, the metallic shells of gold shelled MNPs allow for improved photothermal responses [20], improved X-ray contrast in computer tomography (CT) imaging [21] and improved magnetic resonance imaging (MRI) contrast characteristics [22], which can be ultimately combined with magnetically responsive cores (MHT, CT and MRI) for applications in theranostics [21,23]. It has been reported that the MHT performance (i.e., steady state condition (SSC) with short transient times and asymptotic temperatures in the surrounding environment) of gold shelled MNPs decreases as the thickness of the gold shell increases, which has been claimed to be due to the interaction at the shell–core interface and the loss of cooperative behavior between neighboring MNPs [23]. Interestingly, as strongly suggested in the present report, the fine tuning of the shape and size of the magnetic cores and the thickness of the metallic shells and their composition seems to provide a way to modulate $T_{\infty }$ in MHT protocols, i.e., the typical time that is needed for the system (a host medium that incorporates the metal shelled MNPs) to achieve $T_{\infty }$ and the value of $T_{\infty }$ itself. The impact of the size and shape of MNPs on MHT performance has been explored in recent years [24,25].

The present study investigated the successful modulation of $T_{\infty }$ in MHT protocols via the modulation of the thickness of the gold shells that were deposited onto maghemite NPs (with a fixed particle size and particle concentration) under different external conditions for the AC (alternating current) applied magnetic fields. Asymptotic temperatures that were in the range that is required for MHT to trigger cellular apoptosis were achieved for magnetic field frequencies and field amplitudes that were in the range of 100–650 kHz and 9–25 mT, respectively. Moreover, the time (t) dependence of the system’s temperature (T) was recorded up to the SSC and curve-fitting using a mathematical function that was developed in this study, which allowed for the extraction of τ (typical heat transfer time) and $T_{\infty }$ from the achieved SSC. Importantly, the successful modulation of the SSC in the MHT protocols represented robust proof that the pre-setting of certain parameters for application in cellular apoptosis was actually possible. Additionally, the presented mathematical derivation of the function that was used to model the temperature increases during MHT was pioneering and extended the similar phenomenological mathematical expression that has been long employed in the literature, i.e., the Box–Lucas method [26,27,28,29,30]. At this stage, it is worth mentioning that the Box–Lucas method has also been used to describe temperature increases that are due to photothermal effects [31,32]. An important metric for the application of MHT technology is the so-called specific absorption rate (SAR), which is defined as the thermal power that is dissipated per unit mass (kW/kg). A key piece of information that is required to assess the SAR value is the initial slope of the T versus t curve. A huge variety of magnetic nanomaterials (with varying morphologies and phases) has been tested for use in MHT and the corresponding SAR values have been reported in the literature. SAR values in the range of 10 to 300 kW/kg have been reported for spherical iron oxide NPs [33,34,35,36]. Different shapes and sizes of magnetic NPs have also been tested in regard to their performance in MHT technology and have produced SAR values in the range of 50 to 1000 kW/kg [37]. Importantly, different configurations of core–shell magnetic NPs have also been tested for MHT performance and have produced SAR values in the range of 60 to 400 kW/kg [38,39,40]. Finally, gold shelled core–shell spherical magnetic NPs (CoFe_2_O_4_@Au) have been tested for MHT performance and have produced SAR values in the range of 10 to 55 kW/kg [41]. Although gold shelled core–shell magnetic nanoparticles produce smaller SAR values (typically one order of magnitude smaller) compared to their gold unshelled counterparts, there is an increasing interest in developing this morphology for MHT. As well as protecting the magnetic cores against leaching, gold shelled core–shell magnetic NPs provide very unique surface functionalization capabilities, which allow for the easy anchoring of a range of bioactive molecules [42,43].

We envisage that the present report could contribute to the opening of a new chapter on breakthroughs in MHT technology by providing a new strategy for safely promoting cell apoptosis using thermotherapy, which could help MHT to achieve the status of a safe technology for use in practice. Last but not least, photothermal therapy could also benefit from the material strategy and analysis that are reported in this paper.

## 2. Materials and Methods

**Figure 1:**
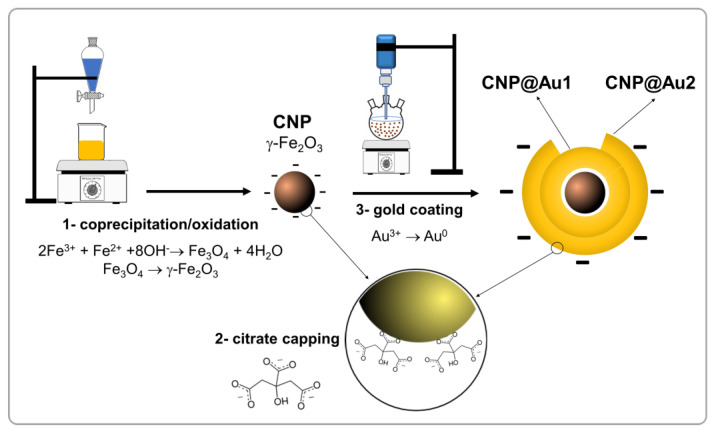
A diagram of the synthesis of the citrate-capped (CNP) and gold shelled (CNP@Au1 and CNP@Au2) maghemite NPs.

The gold shelled maghemite NPs were prepared using a slightly modified procedure (upscaling) that was described by Coelho et al. [19] and is summarized in Figure 1. Firstly, maghemite (γ-Fe_2_O_3_) NPs were obtained from the coprecipitation of magnetite (Fe_3_O_4_) NPs via acidic oxidation. Briefly, the protocol for coprecipitating the maghemite NPs comprised the use of 50 mL of solution (0.10 mol/L Fe^3+^, 0.05 mol/L Fe^2+^ and 0.10 mol/L HCl), which was stirred with 250 mL of 1.0 mol/L NH_4_OH for 30 min. After washing (five times with water and once with 0.5 mol/L HNO_3_), the obtained precipitate was boiled for 30 min with 0.50 mol/L Fe (NO_3_)_3_. The magnetic precipitate was then separated with a permanent magnet and stirred with trisodium citrate solution (1.0 mol/L) at 80 °C for 30 min (with a molar ratio of citrate to iron of 0.1) in order to fabricate citrate-capped maghemite NPs. The obtained magnetic precipitate was washed with acetone, redispersed in water (with the pH adjusted to ~7.4) and labeled as sample “CNP”. Secondly, to obtain the gold shelled maghemite NPs, 320 mg of the citrate-capped maghemite NPs (CNP) was dispersed in 500 mL of water under ultrasonication. Then, reagents were added in the following HAuCl_4_/NaBH_4_ sequence: 1.8 mL of 1.0 wt% aqueous HAuCl_4_ (10 min sonication); 1.5 mL of 0.3 mol/L ethanolic NaBH_4_ (10 min sonication). Four cycles of the HAuCl_4_/NaBH_4_ sequence were employed to prepare the first gold shelled maghemite NP sample (CNP@Au1) and eight cycles of the HAuCl_4_/NaBH_4_ sequence were performed to prepare the second gold shelled maghemite NP sample (CNP@Au2). The final dispersions that contained the gold shelled maghemite NPs started with the previously prepared samples (CNP@Au1 and CNP@Au2), which were then concentrated in a rotary evaporator, washed and redispersed in water at pH ~7.4 with an Fe concentration of ~40 mg Fe/mL. It is worth mentioning that the three investigated samples comprised the same maghemite core: gold unshelled maghemite (CNP); gold shelled maghemite with a thin gold shell (CNP@Au1); and gold shelled maghemite with a thick gold shell (CNP@Au2).

The crystalline structures and the average size of the synthesized maghemite NPs were analyzed in an X-ray Miniflex 600 diffractometer (Rigaku, Tokyo, Japan) using CuKα radiation (λ = 1.541 Å) at 40 kV and 30 mA. For the X-ray diffraction (XRD) evaluation, the samples were deposited onto the surface of a zero-background sample holder. The average crystallite size of the maghemite NPs was estimated using Scherrer’s formula for the broadening of the most intense XRD peak (311). Additionally, the morphology and size distribution of the maghemite NPs were assessed by transmission electron microscopy (TEM) using a JEM-2100 (JEOL, Akishima, Japan) microscope, which operated at 200 kV and was equipped with energy dispersive spectroscopy (EDS) from Thermo Scientific (Thermo Scientific, Waltham, MA, USA). The samples were dispersed in ultrapure water under ultrasonication and were further deposited onto a lacey carbon-supported copper grid. Zetametry was employed to determine the zeta potential of the maghemite NPs, which was estimated through electrophoretic experiments using a Nano ZS ZetaSizer with a DTS 1070 disposable cuvette (Malvern Panalytical, London, UK). The results of the electrophoretic mobility were converted into zeta potential values using Henry’s equation [44]. The gold and maghemite contents of the samples were determined through inductively coupled plasma optical emission spectrometry (ICP-OES) using a Perkin Elmer Optima 8000 (Perkin Elmer, Waltham, MA, USA) [45]. The gold/maghemite (g/m) ratios were obtained for samples CNP@Au1 (0.0376) and CNP@Au2 (0.0752). The room temperature magnetization curves were recorded through vibrating sample magnetometry (VSM) using the ADE Technologies model EV7 instrument (Microsense LLC, Lowell, MA, USA).

To evaluate the heating performance of the synthesized magnetic samples, 1-mL aliquots of the corresponding aqueous solutions (CNP, CNP@Au1 and CNP@Au2), which all contained 40 mg Fe/mL, were introduced into vials and placed in the coil center of the MHT apparatus, which was a magneTherm system (nanoTherics Ltd., Dig Lane, UK). The temperature profiles were monitored with a fiber optic thermometer (LumaSense Technologies Inc., Denver, CO, USA) fiber-optic thermometer during the MHT procedure. Five different frequencies of the MHT apparatus were tested (112, 167, 334, 473 and 631 kHz). Likewise, five alternate magnetic field amplitudes were tested in combination with the above-mentioned frequencies (9, 11, 17, 18 and 25 mT).

## 3. Results and Discussion

### 3.1. Sample Characterization

**Figure 2:**
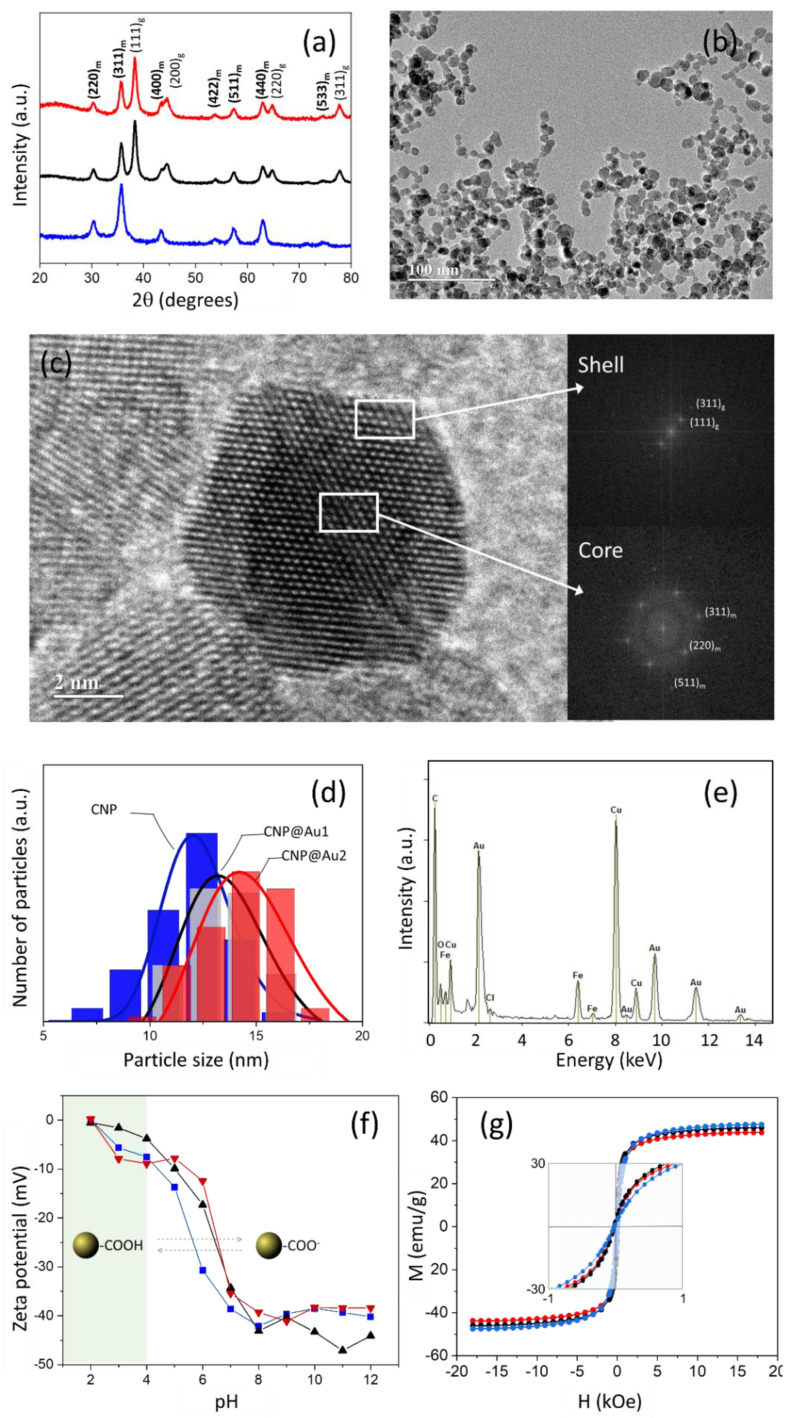
(**a**) The X-ray diffraction patterns of CNP (blue), CNP@Au1 (black) and CNP@Au2 (red), in which the main diffraction peaks of the maghemite (m) phase are indicated by the red pattern (30.3° (220), 35.6° (311), 43.3° (400), 53.7° (422), 57.3° (511), 62.9° (440) and 74.5° (533)) and the main diffraction peaks of the metallic gold (g) phase are indicated by the red pattern (38.3° (111), 44.4° (200), 64.6° (220) and 77.7° (311)); (**b**) a typical TEM image of the gold shelled sample (CNP@Au2); (**c**) an HRTEM image of a single nanoparticle from CNP@Au2 (left-hand side), which emphasizes spots (white rectangles) in the shell and core regions, and the corresponding FFT analysis (right-hand side); (**d**) the size distributions (histograms) of the NPs in CNP (blue), CNP@Au1 (gray) and CNP@Au2 (red); (**e**) a typical HRTEM/EDS spectrum of a selected region in CNP@Au2; (**f**) the zeta potential as a function of the pH for CNP (blue squares), CNP@Au1 (black triangles) and CNP@Au2 (red triangles); (**g**) the room temperature hysteresis loops of CNP (blue), CNP@Au1 (black) and CNP@Au2 (red) and the corresponding magnetization at reduced field range (inset). The solid lines in (**f**,**g**) are only presented as a guide.

Figure 2a shows the XRD patterns of the synthesized samples (CNP, CNP@Au1 and CNP@Au2) and indicates the main XRD peak positions of the maghemite (JCPDS 39–1346) and metallic gold (JCPDS 04-0784). Only XRD peaks for the maghemite phase were observed in the CNP sample (gold unshelled). In contrast, in the gold shelled maghemite NPs (CNP@Au1 and CNP@Au2), a sub-group of XRD peaks for metallic gold (g) could be observed as well as the maghemite XRD peaks (m). The average crystallite sizes of the magnetic cores ($d_{\mathrm{XRD}}$), which were calculated from the 311 XRD peak (maghemite phase) using Scherrer’s formula, are listed in Table 1. It is worth mentioning that Scherrer’s formula is given by $d_{\mathrm{XRD}}=K\lambda /\beta \cos\theta$, where $d_{\mathrm{XRD}}$ is actually correlated with the average volumetric size (average diameter) of the crystallite and K, λ, β and θ represent the shape factor (which is equal to 0.9 for spherical NPs), the employed X-ray wavelength, the corrected XRD line broadening at half of the maximum intensity (FWHM) and the Bragg angle, respectively [46]. Using the 311 XRD peak, the extracted $d_{\mathrm{XRD}}$ values (see the first row in Table 1) indicated that the average size of the magnetic cores was fairly preserved following gold shelling as there were no significant differences between the $d_{\mathrm{XRD}}$ values of the gold unshelled (CNP: $d_{\mathrm{XRD}}=11.8\;\mathrm{nm}$) and gold shelled (CNP@Au1: $d_{\mathrm{XRD}}=12.3\;\mathrm{nm}$; CNP@Au2: $d_{\mathrm{XRD}}=12.1\;\mathrm{nm}$) samples.

All of the synthesized NPs, gold unshelled as well as gold shelled, presented nearly spherical morphologies (as shown in Figure 2b, which presents a typical TEM micrograph of CNP@Au2). Figure 2c shows a high-resolution transmission electron microscopy (HRTEM) image of a single gold shelled maghemite NP and clearly demonstrates the synthesis of the spherical core–shell nanostructures. The fast Fourier transform (FFT) analysis of the HRTEM image was carried out on a single particle and assessed the spot diffraction patterns of both the core and shell regions (see the white rectangles in Figure 2c). The data analysis indicated that the spots in the shell region corresponded to the (111) and (311) atomic planes of the cubic phase of the metallic gold, with interplanar distances of 0.24 nm and 0.12 nm, respectively. Likewise, the spots in the core region corresponded to the (111), (311) and (511) atomic planes of the spinel phase, with interplanar distances of 0.48 nm, 0.25 nm and 0.16 nm, respectively. The revealed crystal structures were also supported by the XRD data and confirmed the successful formation of the γ-Fe_2_O_3_/Au core–shell structures, as assessed using the HRTEM images.

The associated size histograms (vertical bars) of the citrate-capped (CNP) and gold shelled (CNP@Au1 and CNP@Au2) maghemite NPs, which were drawn from the recorded TEM images and the corresponding curve-fitting (solid lines) using a log-normal distribution function, are shown in Figure 2d. It is worth mentioning that the functional form of the log-normal distribution function that was used to fit the particle size histograms that were extracted from the TEM micrographs was: $P\left(d\right)={\left(d\sigma_{\mathrm{TEM}}\sqrt{2\pi }\right)}^{-1}\exp\left[-{\left(\ln d/d_{\mathrm{TEM}}\right)}^{2}/2\sigma_{\mathrm{TEM}}^{2}\right]$, where $d_{\mathrm{TEM}}$ and $\sigma_{\mathrm{TEM}}$ are the mean diameter (logged data) and standard deviation (logged data), respectively [47]. The parameters that were extracted from the histogram curve fitting are summarized in Table 1. Although the mean diameter ($d_{\mathrm{TEM}}$) and standard deviation ($\sigma_{\mathrm{TEM}}$) of the gold shelled maghemite NPs in the TEM images were very much the same, they were slightly larger than the mean diameter ($d_{\mathrm{TEM}}$) and standard deviation ($\sigma_{\mathrm{TEM}}$) of the gold unshelled maghemite NPs (see Table 1). Importantly, the mode (i.e., the particle size that corresponded to the global maximum of the histogram curve) of the solid lines (blue, black and red) that are displayed in Figure 2d systematically shifted to higher values as the g/m ratio increased, which strongly suggested the growth of the gold shell layers at the increasing thickness between the CNP@Au1 and CNP@Au2 samples. The HRTEM/EDS spectrum of the CNP@Au2 sample, as shown in Figure 2e, revealed peaks that corresponded to Au and Fe, which also supported the claim that the MNPs in CNP@Au1 and CNP@Au2 presented γ-Fe_2_O_3_-Au biphasic structures.

In addition to the above assessment of particle size using both the XRD and TEM techniques, we further deepened the analysis by correlating the values that were extracted using the two employed techniques. In this regard, the average crystallite size was assessed using Scherrer’s equation scales for the mean diameter ($d_{\mathrm{TEM}}$) and standard deviation ($\sigma_{\mathrm{TEM}}$) values that were extracted from the analysis of the TEM particle size histograms via $d_{\mathrm{XRD}}^{\mathrm{TEM}}=\left(3d_{\mathrm{TEM}}/4\right){\left[\exp{\left(\sigma_{\mathrm{TEM}}\right)}^{2}\right]}^{7/2}$ [48]. It is important to stress that $d_{\mathrm{XRD}}^{\mathrm{TEM}}$ represents the average crystallite size of the magnetic cores, which were estimated using the parameters that were extracted from the curve fitting of the particle size histograms (see Figure 2d). In Table 1, the second row shows the values of $d_{\mathrm{XRD}}^{\mathrm{TEM}}$ that were calculated using the values in the third and fourth rows. A more realistic comparison of the average sizes could then be performed by looking at the values in the first and second rows of Table 1. Still, the values of $d_{\mathrm{XRD}}^{\mathrm{TEM}}$ were systematically slightly above the values of $d_{\mathrm{XRD}}$. It is worth mentioning that majority of the data that have been reported in the literature have shown $d_{\mathrm{TEM}}$ values that were larger than the $d_{\mathrm{XRD}}$ values [49]. In the literature, this discrepancy has usually been attributed to the presence of an amorphous dead layer (which leads to the well-known magnetic dead layer in magnetic nanomaterials) at the surface of nanomaterials, which is not picked up by XRD measurements but is captured in TEM micrographs [50,51]. Therefore, it was quite clear that our comparison of particle sizes (XRD versus TEM) should be carried out using $d_{\mathrm{XRD}}^{\mathrm{TEM}}$ and $d_{\mathrm{XRD}}$ data instead of $d_{\mathrm{TEM}}$ and $d_{\mathrm{XRD}}$ data. Then, in line with the above-presented arguments, the slightly larger values of $d_{\mathrm{XRD}}^{\mathrm{TEM}}$ ($d_{\mathrm{XRD}}^{\mathrm{TEM}}≳d_{\mathrm{XRD}}$), as observed in Table 1 (first and second rows), could be reasonably credited to the surface amorphous dead layer. Moreover, it is worth mentioning that the $d_{\mathrm{XRD}}^{\mathrm{TEM}}/d_{\mathrm{XRD}}$ ratio increased monotonically between CNP (about 0.8%) and CNP@Au1 (about 6.5%) and CNP@Au2 (about 11.6%). It was quite obvious that this trend was due to the increasing thickness of the gold shell layer, which was not picked up while using Scherrer’s formula to extract the $d_{\mathrm{XRD}}$ values from the 311 XRD peak of the maghemite phase. This observation provided extra evidence for the successful fabrication of the reported core–shell structures.

Figure 2f shows that the zeta potential of the synthesized MNPs was strongly pH-sensitive and varied from 0 mV to −45 mV within the investigated range of pH (2–12). As expected for the CNP sample, this finding could be associated with the deprotonation of the –COOH groups (from the adsorbed citrate at the surface of the MNPs) as the pH increased, which reflected the enhancement of the negative zeta potential values due to the increasing number of negatively charged surface groups [52]. Additionally, the gold deposition onto the citrate-capped maghemite NPs yielded gold shelled MNPs with citrate molecules attached to their surface [19]. Thus, as shown in Figure 2f, the zeta potential of the gold shelled MNPs was also highly negative at physiological pH values and did not differ significantly from the value that was found for the CNP sample. Moreover, these high values of negative zeta potential indicated the increasing stability against NP aggregation that was due to particle–particle electrostatic repulsion. It is generally assumed that zeta potential values of higher than 30 mV provide stable conditions in charged colloids [53]. This finding corroborated the experimental observations that the solutions of the fabricated MNPs were very much stable with negligible zeta potential variation after months of storage.

As shown in Figure 2g, all of the maghemite-based synthesized samples (CNP, CNP@Au1 and CNP@Au2) displayed room temperature superparamagnetic behavior with negligible remanence and coercivity (see the inset). Moreover, the saturation magnetization of the samples decreased as the nonmagnetic gold shell thickness increased (see Table 1), which was in good agreement with the literature [15]. Impressively, the saturation magnetization of the CNP, CNP@Au1 and CNP@Au2 samples (last row in Table 1) could be analyzed against the relative g/m contents (ICP-OES data) that were obtained for CNP@Au1 (0.0376) and CNP@Au2 (0.0752). Assuming that all three samples had the same magnetic core (48.5 emu/g), the reductions in the saturation magnetization that was due to the increasing gold content was estimated to be about 47 and 45 emu/g for CNP@Au1 and CNP@Au2, respectively. These findings could then be compared to the values that were recorded from the magnetometry (VSM), as shown in Table 1, i.e., about 46 and 44 emu/g for CNP@Au1 and CNP@Au2, respectively. The two sets of data revealed an impressive consistency between the values that were independently recorded from two different experimental techniques (ICP-OES and VSM).

### 3.2. Magnetic Hyperthermia Data Analysis

The so-called Box–Lucas method is claimed to be a phenomenological model for fitting the time (t) dependence of the temperature (T) of a liquid medium that contains MNPs in suspension and is under the influence of an externally applied alternating (AC) magnetic field [26,27,28,29,30]. This data fitting approach has been systematically reported in the literature as being the most suitable method for modeling experimental T versus t data [29]. However, to the best of our knowledge, the present study demonstrated for the first time that the Box–Lucas method emerged naturally (instead of phenomenologically) from the definition of two well-established concepts. First, the thermal current ($H_{S}=dQ/dt$), which flows across the interface between the MNPs and the outside liquid medium (i.e., the surrounding environment) and transfers heat (Q) at a given rate (dQ/dt) from the hot core of the MNPs to the colder liquid medium. Second, the thermal conductivity ($k_{S}$) of the MNP interface (gold shell) with the liquid medium, which is given by $k_{S}=-H_{S}/A\times \left(dT/dr\right)$, where A is the area that is perpendicular to the heat flow direction (r) at a given temperature gradient (dT/dr). Therefore, the thermal current that flows outward across the gold shell layer was given by:

(1) $$H_{S}=-k_{S}\;A\times \left(dT/dr\right).$$

**Figure 3:**
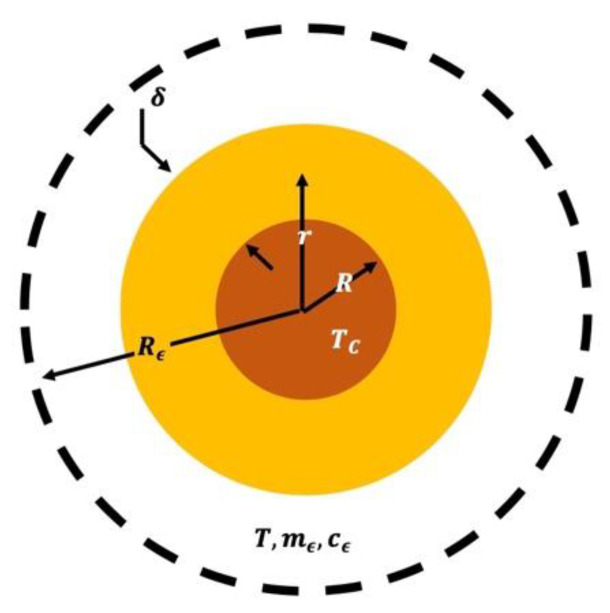
A schematic representation of a single spherical shelled MNP when dispersed in a liquid medium (surrounding environment) with an average nanoparticle concentration scaling of $3/4\pi R_{\epsilon }^{3}$.

In order to move forward from Equation (1), we needed to take into account the morphology of the MNPs, i.e., their shapes and physical dimensions. Then, based on the recorded HRTEM micrographs (see Figure 2c, for instance), we took the simplest case of a spherical core–shell system with a magnetic core of radius R that was shelled by a nonmagnetic shell (interface) of thickness δ and thermal conductivity $k_{S}$, as schematically shown in Figure 3. Starting from Equation (1) and taking the spherical surface area of radius r as $A=4\pi r^{2}$, Equation (2) was obtained:

(2) $$H_{S}\frac{dr}{r^{2}}=-4\pi k_{S}\;dT.$$

By assuming the SSC of heat transfer with a constant thermal current ($H_{S}$), Equation (2) could be integrated into the shell thickness (δ), i.e.:

(3) $$H_{S}\int_{R}^{R+\delta }\frac{dr}{r^{2}}=-4\pi k_{S}\int_{T_{C}}^{T}dT,$$

where $T_{C}$ is the average core temperature and T ($T<T_{C}$) is the average outside temperature that extends to a radius of $R_{\epsilon }$ (see Figure 3). After integrating Equation (3), we obtained:

(4) $$H_{S}\left(\frac{1}{R}-\frac{1}{R+\delta }\right)=4\pi k_{S}\left(T_{C}-T\right).$$

The thermal current ($H_{S}=dQ/dt$) was now substituted into Equation (4) to provide:

(5) $$\frac{dQ}{dt}=4\pi k_{S}\frac{R\left(R+\delta \right)}{\delta }\left(T_{C}-T\right).$$

By integrating $dQ=m_{\epsilon }c_{\epsilon }dT$ into Equation (5), where $m_{\epsilon }$ and $c_{\epsilon }$ are the average values of the mass and specific heat of the suspension within the region of radius $R_{\epsilon }$ (surrounding medium), respectively, we obtained:

(6) $$m_{\epsilon }c_{\epsilon }\frac{dT}{dt}=4\pi k_{S}\frac{R\left(R+\delta \right)}{\delta }\left(T_{C}-T\right),$$

which could be rearranged to provide:

(7) $$\frac{dT}{dt}=\frac{\left(T_{C}-T\right)}{\tau }\;,$$

where the typical heat transfer time (τ) is given by $\tau =\left(m_{\epsilon }c_{\epsilon }/4\pi k_{S}\right)\left[\delta /R\left(R+\delta \right)\right]$.

The solution to Equation (7) was simply the so-called Box–Lucas method, which was obtained from very well-established concepts regarding thermal transfer across the interface between different media, i.e., the core of the MNPs and the liquid medium. Importantly. By taking this route to obtain Equation (7), we found a relationship between the typical heat transfer time (τ) and the characteristics of the interface ($k_{S}$), which could be ultimately engineered during the synthesis protocol. It is worth mentioning that the relationship between τ and $k_{S}$ was inverse, meaning that in a typical MHT experiment, metal shelled MNPs would be expected to achieve the SSC later than unshelled MNPs. Additionally, the characteristics of the surrounding medium ($m_{\epsilon }$ and $c_{\epsilon }$) were included in Equation (7) as well. Impressively, the solution to Equation (7) unveiled a relationship between the typical heat transfer time (τ) and the morphological (shape and size) aspects of the MNPs, as well as the thickness of the metal shell. The metal shell thickness and the shape and size of the MNPs were extremely important to consider while engineering the MNPs in order to meet specific requirements of the heat transfer time (τ). The claimed phenomenological model of the Box–Lucas method failed to unveil all of the key aspects that were emphasized by the heat transfer time (τ). Finally, the solution to Equation (7) provided (analytically) the well-known Box–Lucas method:

(8) $$T\left(t\right)=T_{\infty }\left[1-\eta e^{-t/\tau }\right]\;\;\;\;;\;\;\;\eta =\left(T_{\infty }-T_{0}\right)/T_{\infty }\;,$$

where $T_{0}$ is the average initial temperature of the liquid medium and $T_{\infty }$ is the average asymptotic temperature (@ t→∞) of the SSC for heat transfer. Notice that in Equation (8), $T_{\infty }\to T_{C}$ as t→∞.

**Figure 4:**
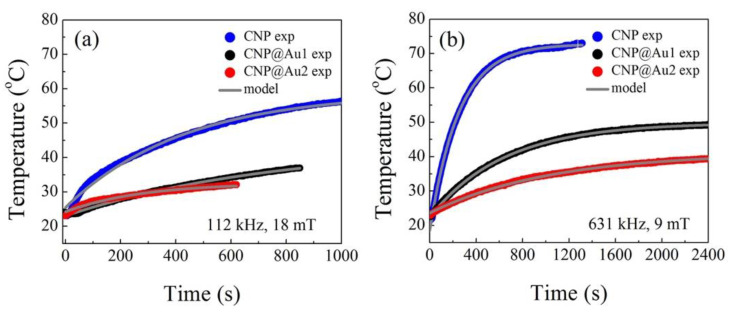
The temperature (°C) versus time (s) curves of the aqueous suspensions that contained MNPs (CNP, CNP@Au1 and CNP@Au2) and were under the influence of an externally AC magnetic field: (**a**) the data from an AC magnetic field amplitude of 18 mT and frequency of 112 kHz for CNP, CNP@Au1 and CNP@Au2; (**b**) the data from an AC magnetic field amplitude of 9 mT and frequency of 631 kHz for CNP, CNP@Au1 and CNP@Au2. The experimental data are represented by filled symbols and the fitting data are represented by solid gray lines.

It is worth mentioning that a very important piece of information was extracted from the fitting of the T versus t data (see Figure 4), namely the initial slope of the T versus t curve, i.e., ${\left(dT/dt\right)|}_{t\to 0}$. The initial slope was used to define the SAR, which could be estimated using $SAR=c\left(m_{\epsilon }/m_{s}\right){\left(dT/dt\right)|}_{t\to 0}$, where c, $m_{\epsilon }$ and $m_{s}$ are the solvent’s specific heat capacity, mass of suspension and mass of solution (nanoparticles), respectively [54]. Although ${\left(dT/dt\right)|}_{t\to 0}$ was extracted from the fitted data in practice, it should be kept in mind that Equation (8) could be used to find ${\left(dT/dt\right)|}_{t\to 0}=\left(4\pi k_{s}/m_{\epsilon }c_{\epsilon }\right)\left[R\left(R+\delta \right)/\delta \right]\left(T_{\infty }-T_{0}\right)$. This was an interesting aspect when correlating the SAR values with the typical shapes and sizes of the NPs (e.g., R and δ in Figure 3). Within the context of the present discussion, we found that $SAR≅\left(4\pi k_{s}/m_{s}\right)\left(T_{\infty }-T_{0}\right)\left[R\left(R+\delta \right)/\delta \right]$, as long as $c≅c_{\epsilon }$.

### 3.3. Fitting Procedure for T Versus t Data

Equation (8) was used to fit the collected T versus t data, as presented in Figure 4. Figure 4 also shows the representative data (filled symbols) and the corresponding fitting data (solid gray lines) that were obtained using Equation (8).

Table 2 shows the parameters (τ, $T_{\infty }$ and $T_{0}$) that were obtained from the fitting data for all of the performed MHT experiments, with the typical average heat transfer time (τ) in seconds (s) and the average values of $T_{\infty }$ and $T_{0}$ in degrees Celsius (°C). Moreover, the last column of Table 2 includes the estimated values of the specific absorption rate (SAR), which were extracted from the T versus t data. Notice that Table 2 also includes the characteristics of the externally applied alternating (AC) magnetic field, with field amplitude (H) in mT and frequency (f) in kHz. In this regard, it was important to compare the SAR data of the gold shelled γ-Fe_2_O_3_ NPs (see Table 2), which varied in the range of 2 to 75 kW/kg, to the SAR data that were reported by Sabale et al. [41] for gold shelled CoFe_2_O_4_ NPs, which varied in the range of 10 to 55 kW/kg. The highest SAR value that was achieved for gold shelled CoFe_2_O_4_ NPs was about 55 kW/kg (experimental conditions: 5 mg CoFe_2_O_4_/mL, 276 kHz and 34 mT). In contrast, the highest SAR value that was achieved for gold shelled γ-Fe_2_O_3_ NPs (present report) was about 75 kW/kg (experimental conditions: 40 mg Fe/mL, 334 kHz and 17 mT). Actually, the 5 mg CoFe_2_O_4_/mL represented 2.4 mg Fe/mL. Therefore, by considering the inverse relationship of $SAR\propto \left(1/m_{s}\right)$ and neglecting any changes in the remaining parameters that were associated with SAR, we could estimate the SAR values for gold shelled CoFe_2_O_4_ NPs with 40 mg Fe/mL, which were roughly equal to: SAR(40 mg Fe/mL)≅SAR(2.4 mg Fe/mL)×(2.4/40)≅55×(2.4/40)≅3.3kW/kg. So, roughly speaking, the gold shelled γ-Fe_2_O_3_ NPs in this study presented the best SAR values (see Table 2: 75 kW/kg), which were about twenty times ((75/3.3)≅20. ) bigger than the best SAR values that have been reported for gold shelled CoFe_2_O_4_ NPs [41].

**Figure 5:**
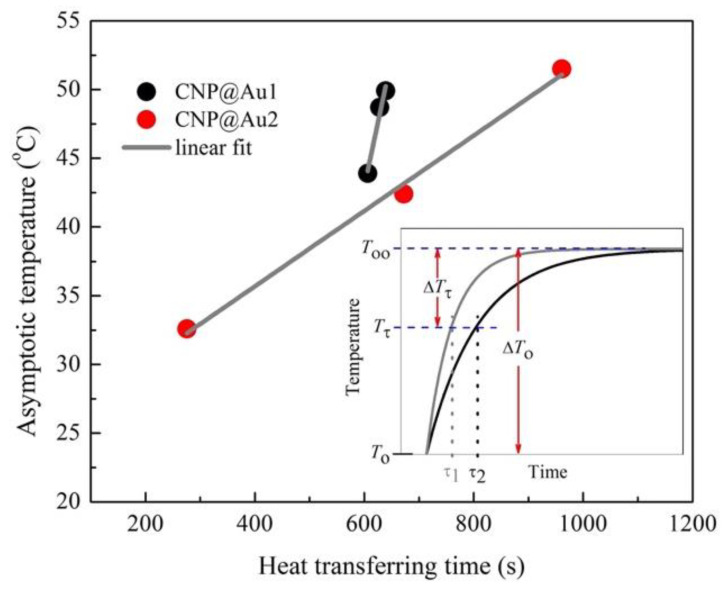
The asymptotic temperature ($T_{\infty }$) in degrees Celsius (°C) versus the typical heat transfer time (τ) in seconds for selected $T_{\infty }$ versus $\tau_{\mathrm{CNP}@\mathrm{Au1}}$ (dark gray boxes in Table 2) data (black circles) and $T_{\infty }$ versus $\tau_{\mathrm{CNP}@\mathrm{Au2}}$ (light gray boxes in Table 2) data (red circles). The inset shows a schematic diagram of the relationship between $\Delta T_{0}$ and $\Delta T_{\tau }$, i.e., $\Delta T_{\tau }=\Delta T_{0}/\delta ≅37\%\Delta T_{0}$.

At this point, it was important to derive an expression for the ratio between the typical heat transfer time ($\tau =\left(m_{\epsilon }c_{\epsilon }/4\pi k_{S}\right)\left[\delta /R\left(R+\delta \right)\right]$) that was applied to CNP@Au2 and CNP@Au1, i.e., $\tau_{\mathrm{CNP}@\mathrm{Au2}}/\tau_{\mathrm{CNP}@\mathrm{Au1}}=\left(R+e_{\mathrm{CNP}@\mathrm{Au1}}/R+e_{\mathrm{CNP}@\mathrm{Au2}}\right)\left(e_{\mathrm{CNP}@\mathrm{Au2}}/e_{\mathrm{CNP}@\mathrm{Au1}}\right)$, and the specific mass of both gold (19.32 g/cm^3^) and maghemite (4.88 g/cm^3^) and the relative g/m contents that were obtained (ICP-OES data) for CNP@Au1 (0.0376) and CNP@Au2 (0.0752). These data allowed us to estimate the ratio $\tau_{\mathrm{CNP}@\mathrm{Au2}}/\tau_{\mathrm{CNP}@\mathrm{Au1}}≅1.9\;$. Moreover, this finding ($\tau_{\mathrm{CNP}@\mathrm{Au2}}/\tau_{\mathrm{CNP}@\mathrm{Au1}}≅1.9$) could be compared to the ratio of the average τ-values that were included in Table 2 ($\tau_{\mathrm{CNP}@\mathrm{Au2}}/\tau_{\mathrm{CNP}@\mathrm{Au1}}≅1.4)$, which were extracted from the fitting data of CNP@Au2 and CNP@Au1 under different values of AC magnetic field frequency and amplitude. The fair agreement between the two independent sets of data, i.e., the estimation of the $\tau_{\mathrm{CNP}@\mathrm{Au2}}/\tau_{\mathrm{CNP}@\mathrm{Au1}}\;$ ratio from the assessed ICP-OES data ($\tau_{\mathrm{CNP}@\mathrm{Au2}}/\tau_{\mathrm{CNP}@\mathrm{Au1}}≅1.9$) and the fitting of the T versus t data using Equation (8) ($\tau_{\mathrm{CNP}@\mathrm{Au2}}/\tau_{\mathrm{CNP}@\mathrm{Au1}}≅1.4$), was quite impressive and pointed toward the impact of the thickness of the gold shells that were deposited on top of the maghemite cores when establishing the T versus t curves that were generated by MHT. In addition, the selected $T_{\infty }$ and $\tau_{\mathrm{CNP}@\mathrm{Au1}}$($\tau_{\mathrm{CNP}@\mathrm{Au2}}$) data (dark gray and light gray boxes in Table 2) for similar operating frequencies revealed a linear relationship between $T_{\infty }$ and τ, as shown in Figure 5.

Actually, the linear trend that is shown in Figure 5 emerged from the first derivative of Equation (8), i.e., $dT/dt=\left[\left(T_{\infty }-T_{0}\right)/\tau \right]e^{-t/\tau }$. In the linear regime of the T versus t curve (t≪τ), we had $e^{-t/\tau }≅1$ with the approximately constant dT/dt=η. Therefore, in this limit, $\left(T_{\infty }-T_{0}\right)/\tau ≅\eta$; thus, it was expected that $T_{\infty }$ would scale linearly with τ, i.e., $T_{\infty }≅T_{0}+\eta \tau$, as presented in Figure 5. Finally, the inset of Figure 5 provides a more comprehensive analysis of τ. At the end of the typical heat transfer time (τ), the surrounding medium reached the temperature $T_{\tau }$, which was lower than $T_{\infty }$ by a fixed amount ($\Delta T_{\tau }=T_{\infty }-T_{\tau }≅\eta \tau /\delta$). The $\Delta T_{\tau }$ value scaled with the total temperature change ($\Delta T_{0}=T_{\infty }-T_{0}≅\eta \tau$) via the following relationship: $\Delta T_{\tau }=\Delta T_{0}/\delta$. This means that we could fabricate MNPs to reach pre-set $T_{\infty }$ values at controllable times by modulating τ ($\tau =\left(m_{\epsilon }c_{\epsilon }/4\pi k_{S}\right)\left[\delta /R\left(R+\delta \right)\right]$), e.g., $\tau_{1}<\tau_{2}$ (see the inset of Figure 5), and changing the thermal conductivity of the metallic shells ($k_{S}$) or the shapes and sizes of the MNPs. Once more, our findings confirmed the appropriateness of Equation (7) and its corresponding analytical solution that was represented by Equation (8) (which is known as the Box–Lucas method in the literature) for describing the T versus t curve for the time dependence of the temperature of a liquid medium during MHT.

## 4. Conclusions

In conclusion, the present study investigated the successful modulation of the asymptotic temperature ($T_{\infty }$) of an aqueous suspension that contained gold shelled maghemite nanoparticles, which were tested for applications in magnetic hyperthermia. Importantly, our data showed that $T_{\infty }$ was achieved in less than one hour. Moreover, the typical heat transfer time (τ) could be shortened by engineering the thermal conductivity of the shells and/or the shapes and sizes of the magnetic nanoparticles, which could encourage the exploration of new structures. Importantly, the present study showed that $T_{\infty }$ could be pre-set for increasing gold shell thicknesses and varying operating frequencies and amplitudes of the applied AC magnetic fields. Even more importantly, the achieved values of $T_{\infty }$ could be pre-set to fall within the temperature range that is required to trigger cell apoptosis instead of cell necrosis with a precision of better than 0.3 °C. Impressively, the highest SAR (specific absorption rate) value that was achieved in our experiments (75 kW/kg) was roughly twenty times bigger than the best SAR value that has been reported in the literature for a similar structure. Our findings regarding the modulation of $T_{\infty }$ are expected to provide a breakthrough for establishing clinically safe protocols for magnetic hyperthermia therapy in the near future. Finally, a mathematical expression that described the time dependence of the temperature of a magnetic suspension unveiled the characteristic heat transfer time and its dependence on the shapes and sizes of the core–shell structures. This mathematical derivation, which was based on the well-known concept of heat transfer, was presented for the first time and the derived equation analytically matched the phenomenological equation in the Box–Lucas method, which has been widely used in the literature.

## Figures and Tables

**Table 1:** The morphological and magnetic characteristics of the synthesized samples: $d_{\mathrm{XRD}}$, the average size of the maghemite cores that were extracted from the XRD data; $d_{\mathrm{TEM}}$ and $\sigma_{\mathrm{TEM}}$, the mean diameter and the standard deviation of the MNPs that were extracted from the TEM histograms, respectively; $M_{S}$, the saturation magnetization that was extracted from the hysteresis cycles.

Sample	CNP	CNP@Au1	CNP@Au2
$d_{\mathrm{XRD}}$ (nm)	11.8	12.3	12.1
$d_{\mathrm{XRD}}^{\mathrm{TEM}}$ (nm)	11.9	13.1	13.5
$d_{\mathrm{TEM}}$ (nm)	14 ± 3	15 ± 3	15 ± 3
$\sigma_{\mathrm{TEM}}$	0.19 ± 0.02	0.21 ± 0.02	0.23 ± 0.02
$M_{S}$ (emu/g)	48.5	46.0	43.8

**Table 2:** The parameters (τ, $T_{\infty }$ and $T_{0}$) that were extracted from the fitting of the T versus t data (e.g., see Figure 4) at a fixed combination of the frequency (f) and amplitude (H) of the AC magnetic field. The estimated SAR values (in kW/kg-Fe) are included in last column.

**Sample**	τ (s)	$T_{\infty }\;({}^{\circ}C)$	$T_{0}\;({}^{\circ}C)$	f (kHz)	H (mT)	SAR
CNP	428 ± 4	59.1 ± 0.1	25.5 ± 0.1	112	18	10 ± 2
CNP@Au1	771 ± 6	43.8 ± 0.1	23.1 ± 01	112	18	2.0 ± 0.7
CNP@Au2	276±6	32.6 ± 0.1	24.2 ± 0.1	112	18	4 ± 1
CNP	231 ± 2	72.3 ± 0.2	16.1 ± 0.1	112	25	99 ± 30
CNP@Au1	628 ± 3	48.7 ± 0.1	22.5 ± 0.1	112	25	34 ± 2
CNP@Au2	850 ± 3	43.6 ± 0.1	24.7 ± 0.1	112	25	24 ± 5
CNP	382 ± 3	64.7 ± 0.1	21.4 ± 0.1	167	17	95 ± 20
CNP@Au1	606 ± 6	43.9 ± 0.1	23.0 ± 0.1	167	17	38 ± 5
CNP@Au2	672 ± 7	42.4 ± 0.1	26.3 ± 0.1	167	17	47 ± 10
CNP	216 ± 3	76.5 ± 0.3	21.6 ± 0.3	334	17	177 ± 40
CNP@Au1	559 ± 3	57.7 ± 0.1	27.2 ± 0.1	334	17	75 ± 10
CNP@Au2	961 ± 4	51.1 ± 0.1	25.6 ± 0.1	334	17	36±4
CNP	238 ± 3	74.2 ± 0.2	28.7 ± 0.2	473	11	172 ± 40
CNP@Au1	493 ± 1	57.4 ± 0.1	23.8 ± 0.1	473	11	53 ± 4
CNP@Au2	607 ± 2	47.4 ± 0.1	26.4 ± 0.1	473	11	43 ± 4
CNP	250 ± 1	72.8 ± 0.1	18.5 ± 0.1	631	9	103 ± 20
CNP@Au1	639 ± 1	49.9 ± 0.1	22.5 ± 0.1	631	9	32 ± 1
CNP@Au2	1038 ± 1	41.2 ± 0.1	23.6 ± 0.1	631	9	24 ± 5

## Data Availability

Not applicable.
